# Two-Part Models and Quantile Regression for the Analysis of Survey Data With a Spike. The Example of Satisfaction With Health Care

**DOI:** 10.3389/fpubh.2019.00146

**Published:** 2019-06-11

**Authors:** Odile Sauzet, Oliver Razum, Teresia Widera, Patrick Brzoska

**Affiliations:** ^1^Department of Epidemiology and International Public Health, School of Public Health, Bielefeld University, Bielefeld, Germany; ^2^Centre for Statistics, Bielefeld University, Bielefeld, Germany; ^3^Bundesarbeitsgemeinschaft für Rehabilitation e.V., Frankfurt, Germany; ^4^Health Services Research Unit, Faculty of Health, School of Medicine, Witten/Herdecke University, Witten, Germany

**Keywords:** satisfaction survey, two-part regression models, quantile regression, data with spike, linear regresion

## Abstract

**Background:** Results of patient satisfaction questionnaires can contain a spike at the value corresponding to a complete satisfaction. A possible interpretation is that there are two types of respondents, those who are willing to provide a negative evaluation to one or more items proposed in the questionnaire and those who will always provide a completely positive evaluation irrespective of the item. The aim of the present study is to compare various statistical approaches to the analysis of such data using data from a rehabilitation patient survey of the German Statutory Pension Insurance Scheme as an example.

**Method:** We used data from 272,806 respondents who participated in the survey from 2008 to 2011. We illustrate four models: linear regression, logistic regression, a two-part model based on the assumption of two underlying populations and quantile regression, which does not require any distributional assumptions. For each model we consider the relationship of the satisfaction score with two covariates.

**Results:** While providing correct estimates of the mean values (marginal effects), the assumptions of the linear model are violated which can lead to false interpretations. A two-part regression which consists of a logistic regression followed by a linear regression conditional on not being fully satisfied is a useful alternative. For research questions focusing on specific parts of the distribution, logistic regression as well as quantile regression are to be considered.

**Discussion:** Data with a spike represents a statistical challenge but a range of modeling approaches is available to provide sound interpretations and correct answers to research questions.

## Background

Surveys on the satisfaction of patients, consumers, employees, and other population groups are frequently conducted by various organizations. In the health care setting, they usually consist of a collection of questions concerning, for example, aspects of care, and accommodation. Typically, instruments consisting of several Likert items are employed, on which basis (sub-)scores on different dimensions are calculated. In such surveys, many respondents tend to report complete satisfaction (1–3) resulting in a spike in the distribution of the data at the value corresponding to being fully satisfied.

The presence of a large proportion of a given value of a variable which otherwise follows a known distribution is also common for other health outcomes when there are no restrictive selection criteria. Typical examples also include alcohol or cigarette consumption: a part of the population is abstinent and the rest consumes varying amounts. Various names for this type of data can be found in the literature such as data with “clumping at zero,” with a “spike at zero,” or for count data, zero-inflated. In terms of analysis, such data can pose certain challenges. In observational studies, controlling for confounders or examining multivariate relationships is usually of interest. For a continuous outcome, typically, a linear regression would be performed for that purpose. In the presence of a large proportion of observations having the same value, however, assumptions of linear regression are violated because it is difficult to establish any linear relationship between the outcome (dependent variable) and covariates (independent variables). Moreover, the error term is unlikely to have a normal or any other known distribution. In these cases, also usual techniques like transformation are ineffective in normalizing the data (4). Using linear regression to study associations between outcomes and covariates may therefore lead to biased estimates when applied in this situation. Dichotomising the data to fit a logistic regression model is an option frequently chosen for this scenario. However, it has many disadvantages because all information about the distribution are lost and in regression models relationship between outcome and covariates may disappear (5, 6).

One alternative approach for the analysis of data with a spike is based on the assumption that there are two underlying populations which explain that particular distribution of the data: individuals who are willing to provide a negative evaluation to one or more items proposed in the questionnaire and those who will always provide a completely positive evaluation irrespective of the item (2). Under the two-population assumption, if the outcome is a count (e.g., number of cigarettes), zero-inflated models or hurdle models (7, 8) are widely used. In the case of continuous data (such as satisfaction scores) a two-part model can be applied (9). When such an assumption cannot be made, quantile regression constitutes a feasible approach for the modeling of such data since it lacks any distributional assumptions (10).

Both approaches, a two-part model and quantile regression, may be less well-known to health researchers and the interpretation is not always straight forward. Using a rehabilitation patient survey of the German Statutory Pension Insurance Scheme (11) as an example, we illustrate both approaches and provide an interpretation of the respective regression coefficients obtained. For comparison, we also examine the data by means of linear regression and a logistic regression model and illustrate the shortcomings the application of these methods may have in this situation.

## Methods

### Satisfaction Data

We use data from a cross-sectional representative rehabilitation patient survey implemented as part of a quality assurance program by the German Statutory Pension Insurance Scheme (“Deutsche Rentenversicherung”) among individuals who completed a rehabilitation granted by this organization in one of its rehabilitation clinics (12). The dataset (*n* = 272,806) contains information on patients' satisfaction with different aspects of their rehabilitative treatment. The survey has been conducted during 2008–2011. For our analysis, we use the satisfaction with medical care as an example. Its measurement is based on a composite score calculated by means of three 5-point Likert-scale items on different aspects of medical care with values ranging between 0 (fully dissatisfied) and 5 (fully satisfied). Data is available from the German Statutory Pension Insurance Scheme for researchers who meet the criteria for access to confidential data. Further information (only in German language) are available from http://forschung.deutsche-rentenversicherung.de/FdzPortalWeb/.

For each model we consider the relationship of the satisfaction score with two covariates: age in years and nationality status (German national/non-German national). All analyses were performed with Stata 14 (StataCorp. 2015. Stata Statistical Software: Release 14. College Station, TX: StataCorp LP) and most figures were obtained using the statistical software R (13).

### Two-Part Model

Responses of a population to a satisfaction questionnaire follow a two-part model if the two following conditions are satisfied:

- There is a binomial distribution which determines whether a patient is always going to be fully satisfied (i.e., will never complain about anything) or is willing to complain about something (but may not necessarily do so) and
- Conditional on being willing to complain, the satisfaction score obtained is provided by a specified distribution function.

Giving previous research in the field (2), in the case of satisfaction it is justified to assume that there are two underlying populations responding to the questionnaire; one which will always provide the maximum satisfaction response for all items and one which is willing to provide a negative answer for at least one item. This assumption can be accounted for by a two-part model. The first part of the model consists of a logistic regression, modeling the probability of being fully satisfied. In our example, the model is also adjusted for age and nationality (non-German):

$$\begin{array}{l} Logistic\;(p_{i})=\;\beta_{0}^{logistic}+\;\beta_{1}^{logistic}\;Age_{i}+\beta_{2}^{logistic}nGer_{i} \end{array}$$

The second part of the model, again adjusted for age and nationality, is given by a linear regression model for the satisfaction score *y*_*i*_ conditional on willing to provide a negative answer (i.e., being not fully satisfied):

$$\begin{array}{l} {\text{y}}_{\text{i}}=\beta_{0}^{linear}+\beta_{1}^{linear}\text{Ag}{\text{e}}_{\text{i}}+\beta_{2}^{linear}\text{nGe}{\text{r}}_{\text{i}}\;+e_{i} \end{array}$$

where e_i_ is the error term.

### Quantile Regression

Quantile regression allows, without any distributional assumption, to estimate the parameters of a linear relationship with covariates for the conditional quantile function τ_α(Y)_of an outcomeY,.

$$\begin{array}{l} {\text{τ}}_{\text{α}\left(\text{Y}\right)}\sim \beta_{0}^{\alpha }+\beta_{1}^{\alpha }\text{Ag}{\text{e}}_{\text{i}}+\beta_{2}^{\alpha \;}nGer_{i} \end{array}$$

For example if α = 0.5, a regression model can be obtained for the median of *Y*:

$$\begin{array}{l} {\text{τ}}_{\text{med}}\sim \beta_{0}^{\text{med}}+\beta_{1}^{\text{med}}\text{Ag}{\text{e}}_{\text{i}}+\beta_{2}^{\text{med }}nGer_{i} \end{array}$$

These regression models provide a conditional relationship between the value τ_α(*Y*)_ of satisfaction and covariates (here: age and being non-German). A separate regression model is obtained for each quantile of interest (e.g., 1st quartile, median, 3rd quartile).

Plots of quantile regression parameters were obtained using the Stata command *grqreg* (14).

### Linear and Logistic Regression

To offer a comparison we performed a linear and logistic regression with the same covariates as above. The logistic regression is performed within the two-part model (see above) by dichotomising between fully satisfied (1) and not-fully satisfied patients (0). It basically represents the first part of the two-part model.

## Results

### Sample Description

**Figure 1:**
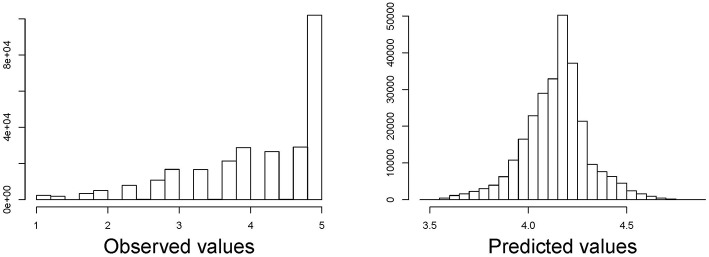
Histogram of satisfaction with medical care score **(left)** and predicted values **(right)** of a linear regression with dependent variables age and nationality (German/non-German).

In total, data on 272,806 individuals was available who had a mean age of 53.3 (SD = 10.1) years. 4.4% of the respondents were non-German. With respect to their satisfaction rating, a large proportion (37.4%) replied to be fully satisfied with their medical treatment (score = 5). The spike at full satisfaction can be seen in the histogram given in Figure 1. The median satisfaction was 4.34.

### Linear Regression

**Figure 2:**
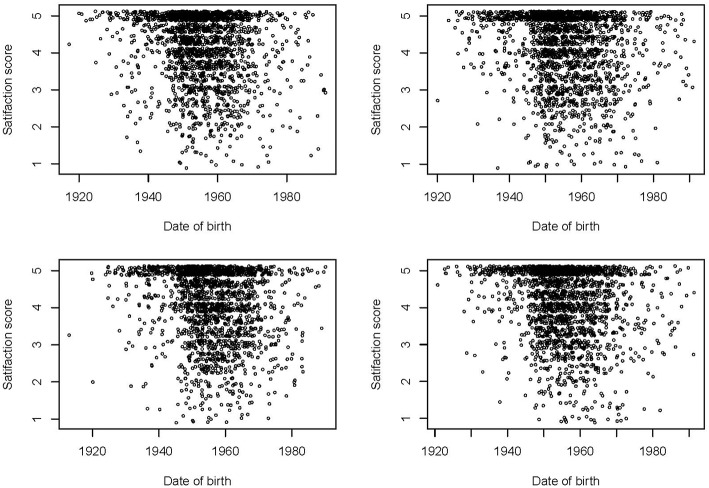
Scatter plots of satisfaction with medical care scores by date of birth in four random sub-samples of 2,000 observations each.

In a linear regression model over the entire distribution, age is positively associated with satisfaction with the score increasing by 0.0164 points per year of age (95%-CI: 0.0161, 0.0168). Non-German, on average, have a 0.064 (95%-CI: 0.047; 0.081) points higher score than German nationals (Table 1). However, the assumption of a linear relationship between age and satisfaction score is not met as illustrated in Figure 2 for four random sub-samples of 2,000 observations each (due to the size of the dataset, it would not be possible to show a scatter plot including all observations). Moreover, the assumption of residuals being normally distributed is violated as can be seen in Figure 1 in which we compare the observed and predicted values for satisfaction by means of residuals obtained from a linear regression model using age and nationality as covariates (residuals are the difference between observed and predicted values). The observed values show a spike at complete satisfaction while the predicted values are almost symmetric. The spike of observations is completely ignored by the model. This indicates that the model does not fit the data at all and highlights the need for an alternative approach.

**Table 1:** Regression coefficients for the linear regression and two-parts model.

	**Regression coefficient**		***N*** **=** **272,806**	
**Model**	**Age**	**95% CI**	**Non-German**	**95% CI**
Linear regression	0.017	[0.016, 0.017]	0.064	[0.047; 0.081]
**Two part model**				
Logistic Regression, OR (being fully satisfied)	1.036	[1.035, 1.037]	1.218	[1.165, 1.273]
Linear regression conditional on possible negative answer	0.0095	[0.0091, 0.0099]	0.0015	[−0.0191, 0.0221]

### Two-Part Regression

Table 1 also provides the regression coefficients resulting from fitting a two-part model.

The first part of the two-part model consists of fitting a logistic regression to model the probability to be fully satisfied (i.e., the data is dichotomised at the value 5). According to the results, the odds of being fully satisfied increases by 3.6% per year of age (OR 1.036, 95% CI [1.035; 1.037]) whereas the odds of being fully satisfied for non-German respondents is 22% higher as compared to German patients (OR 1.218, 95 % CI: [1.173, 1.265]).

The interpretation of the results of the logistic regression in the first part of the model is not sufficient to understand how satisfaction is related to age and nationality across the rest of the distribution. Thus, we move to the second part of the model in which we fit a linear regression to the data under the assumption that one is not fully satisfied. It shows that among those not fully satisfied, on average, increasing age has a positive effect on the satisfaction score. With a coefficient of 0.0095 (95%-CI: 0.0091, 0.0099) the effect size, however, is considerably smaller (non-overlapping confidence intervals) than when a non-conditional linear model is fitted to the entire data (see section Linear regression).

While on average non-German patients are more satisfied than German patients in the non-conditional linear model, the two part model shows that this association is entirely explained by the probability of being fully satisfied. The second part of the model shows that on average the satisfaction of not fully satisfied non-German nationals is not significantly different than the satisfaction of Germans nationals (0.0014, 95% CI [−0.0191, 0.0221]).

### Quantile Regression

**Figure 3:**
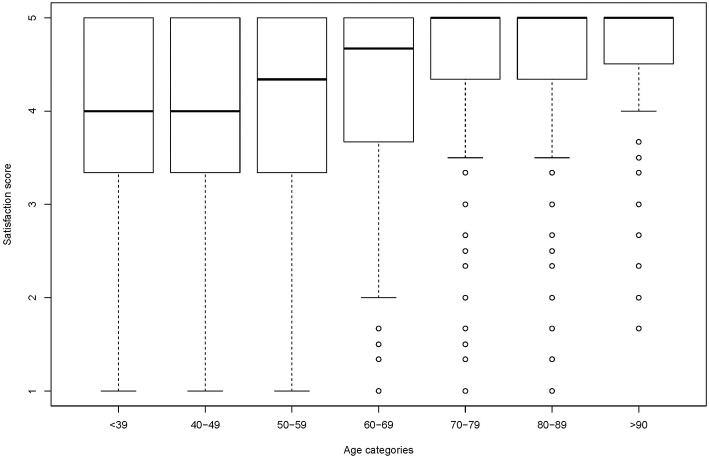
Box plots of satisfaction with medical care per age categories.

Box plots for different age categories can indicate if the relationship between the satisfaction score and age depends on the quantile. In Figure 3, age has been categorized in 7 10-years intervals. The first quartile increases more with age than the median. It can also be seen that for older age categories more than half of the participants are fully satisfied whereas the median decreases with decreasing age. The appearance of box-plots is dependent on the choice of categories and conclusion drawn should be cautious.

Results of the quantile regression are provided in Table 2. A regression model could only be obtained for quantiles up to the median due to the spike at full satisfaction which includes more than 50% of the respondents after a certain age.

**Table 2:** Quantile regression coefficients for age and non-German nationals.

**Quantile**	**Regression coefficient age in year (SE)**	***N* = 272,806 German(0)/non-German(1) (SE)**
0.10	0.025 (0.001)	0.025 (0.026)
0.25	0.027 (0.000)	0.110 (0.014)
0.50	0.022 (0.000)	0.154 (0.015)
0.75	–	–
0.80	–	–

**Figure 4:**
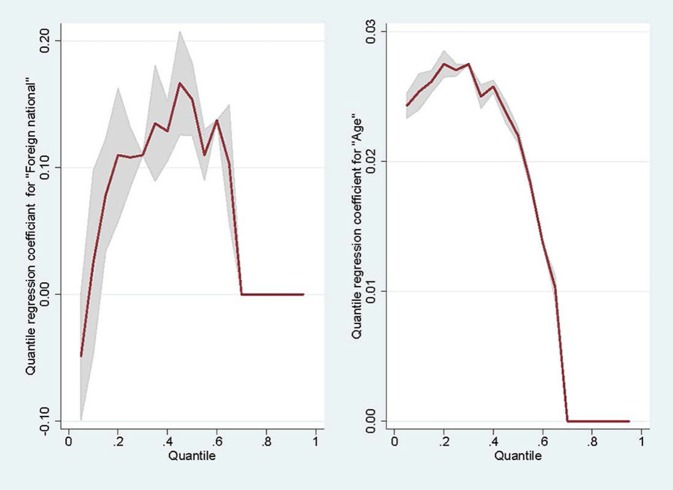
Quantile regression coefficients against quantiles.

Quantile regression shows that there is a positive effect of age for all quantiles which does not vary much between quantiles. This also becomes evident in the plots of quantile regression coefficient per quantile given in Figure 4. This means that the effect of age on satisfaction is constant across the conditional distribution of satisfaction. There is a positive effect of being a non-German national on all quantiles (albeit not being significant on the 0.10 quantile). But this positive effect (the value of the quantile for non-German nationals is higher than for German nationals adjusted for age) increases with the quantile. For example, a non-German needs to have a 0.154 points higher satisfaction score than a German to remain at the median while a patient a year older will need a satisfaction score which is 0.022 points higher to remain at the median conditional on age.

The difference in median satisfaction between non-Germans and Germans is higher than this difference for, say, the 30th percentile. This means that the effect of migration at the top of the conditional distribution of satisfaction is larger than for the lower value of satisfaction.

A way to check for non-misspecification of the model is to verify that the regression lines do not cross along the range of values for age. Given that the intercepts are 1.27, 1.99, and 3.13, respectively, the regression lines for Germans on the one hand and non-Germans on the other hand do not cross for age values ranging from 30 to 100.

## Discussion

When the distribution of an outcome greatly deviates from a normal distribution, an adjusted difference in means between exposure groups or a linear coefficient provide very little information about the relationship between the outcome and the covariates. Using satisfaction with medical care and its relationship with age and nationality as an example, we reviewed two potential alternatives for the analysis of data with a spike at one end of the distribution.

Our analysis illustrates that fitting an (improper) linear model to the data only provides population average values (marginal effects), i.e., in our case, the mean outcome value over all ages in the data and the two nationality groups. Because the assumptions of the linear model are strongly violated, such a model does not allow inferring the true relationship between the outcome score and the covariates.

A two-part model appears to be the more sensible approach in this situation. Using that approach, in our example, first, the probability to be fully satisfied was being modeled and then a linear relationship between the satisfaction score and covariates under the condition of not being fully satisfied was obtained as if there were two underlying populations. Alternatively, a quantile regression provides a linear relationship between any quantile of the distribution of the satisfaction score and covariates. Its advantage is the lack of any distributional assumption. Nonetheless, like in any regression model, a correct specification is necessary to obtain unbiased estimates of regressions coefficients. However, quantile regression is seldom used by health researchers and the interpretation of its findings might be more challenging than for linear and logistic regression coefficients.

Previous research provided evidence showing that a low patient satisfaction is associated with unfavorable rehabilitative outcomes (15, 16). It might be useful to develop interventions to mitigate the effects of factors leading to low satisfaction. While satisfaction increases with age in our study, age has only an effect on the probability of being fully satisfied. Also in terms of differences between Germans and non-Germans, most of the average effect observed in the linear regression disappears if those not fully satisfied are considered. Quantile regression shows that nationality does not actually affect the lowest 10%-quantile of satisfaction. Therefore, any intervention aiming to improve the satisfaction of non-German individuals will not improve the satisfaction of the most dissatisfied. This type of evidence remains neglected by fitting a linear model, but it can be by using a two-part model or a quantile regression.

Modeling continuous data with a spike at zero by means of logistic regression has some major drawbacks because of the loss of information dichotomization incurs. Analyzing dichotomized data only shows a small part of the relationship between the outcome and covariates (17, 18). Therefore, dichotomizing should be done only if there are good reasons to focus on one particular risk group, for example, if it is known that very low satisfaction is associated with increased odds of negative health care outcomes, given a suspected causal link. Distribution-based methods for the dichotomization of continuous outcomes which avoid these drawbacks have been developed. However, they cannot be applied if a spike in the distribution is present (19, 20). They can, though, be applied within the two-part framework for the comparison of groups if the score of the population willing to provide negative answers follows a known distribution. Another alternative to consider would be ordinal logistic regression, for which a larger number of score categories can be constructed (21).

In this paper we focused on satisfaction data with a spike as an outcome and we did not discuss how to model this variable if it is to be used as a covariate in a regression model. This is the object of current research (22, 23). It is also possible to have data with more than one spike and if justified, a three-part model could also be fitted but this adds a new level of difficulties in interpretation and in this case it might be easier to fit a quantile regression instead. Models for longitudinal data with a spike have also been developed (24, 25). Other approaches for data with a spike are discussed in Chang and Pocock (4) and Min and Agresti (9). As mentioned in the introduction, when the outcome is a count, then zero-inflated models should be considered (7, 8).

To our knowledge the use of quantile regression with data with a spike has not been formerly validated and some caution should be applied due to the lack of possibilities to model higher or lower quantiles because of the presence of a spike as this could have an effect on the continuity of residuals.

In this paper we fitted models to illustrate two alternative approaches to the analysis of data with a spike. The simplicity of our analyses with just two covariates means that the associations observed, in particular their direction, should only be considered for illustrative purposes. A more substantive analysis of the determinants of satisfaction and disparities in health care requires to take into account the heterogeneity of the population more thoroughly.

## Conclusion

Using satisfaction with health care as an example, this study illustrated that linear regression can only draw limited evidence about the relationship between the outcome and covariates when the outcome has a spike at one end of its distribution. This is because the assumptions of linearity and normal errors are violated. We have explored alternative models suitable for such data. Under the assumption of two populations generating the data, the simplest and most informative is to use a two-part model. Alternatively or if the two population assumption is not justified, the non-parametric approach offered by quantile regression can be applied.

## Data Availability

The datasets for this manuscript are not publicly available because the data is available from the German Statutory Pension Insurance Scheme for researchers who meet the criteria for access to confidential data. Further information (only in German language) are available from http://forschung.deutsche-rentenversicherung.de/FdzPortalWeb/. Requests to access the datasets should be directed to the above.

## Author Contributions

OS, OR, and PB designed the study. TW provided the data. OS and PB wrote the manuscript. All authors commented and agreed the final version of the version article.

### Conflict of Interest Statement

The authors declare that the research was conducted in the absence of any commercial or financial relationships that could be construed as a potential conflict of interest.
